# Lab-on-a-Chip Platforms for Airborne Particulate Matter Applications: A Review of Current Perspectives

**DOI:** 10.3390/bios12040191

**Published:** 2022-03-24

**Authors:** Sharon Ezrre, Marco A. Reyna, Citlalli Anguiano, Roberto L. Avitia, Heriberto Márquez

**Affiliations:** 1 Instituto de Ingeniería, Universidad Autónoma de Baja California (UABC), Mexicali 21100, Mexico; sharon.ezrre@uabc.edu.mx; 2 Facultad de Ingeniería, Universidad Autónoma de Baja California (UABC), Mexicali 21280, Mexico; rosacitlalli@uabc.edu.mx (C.A.); ravitia@uabc.edu.mx (R.L.A.); 3 Departamento de Óptica, Centro de Investigación Científica y de Educación Superior de Ensenada (CICESE), Ensenada 22860, Mexico; hmarquez@cicese.mx

**Keywords:** airborne particulate matter, Lab-on-a-Chip, particle manipulation, particle monitoring, particle analysis

## Abstract

Lab-on-a-Chip (LoC) devices are described as versatile, fast, accurate, and low-cost platforms for the handling, detection, characterization, and analysis of a wide range of suspended particles in water-based environments. However, for gas-based applications, particularly in atmospheric aerosols science, LoC platforms are rarely developed. This review summarizes emerging LoC devices for the classification, measurement, and identification of airborne particles, especially those known as Particulate Matter (PM), which are linked to increased morbidity and mortality levels from cardiovascular and respiratory diseases. For these devices, their operating principles and performance parameters are introduced and compared while highlighting their advantages and disadvantages. Discussing the current applications will allow us to identify challenges and determine future directions for developing more robust LoC devices to monitor and analyze airborne PM.

## 1. Introduction

Airborne Particulate Matter, or airborne PM, are small particles and liquid droplets whose presence in the air is considered a global issue, since it contributes to serious pollution effects among exposed populations [1]. Airborne PM exhibits a complex chemical and physical heterogeneity between organic and inorganic components in concentrations that fluctuate over time and space in consequence of climatological variations and chemical reactions between pollutants and atmospheric compounds [2]. Due to their complex physicochemical differences, airborne particles are classified by their Equivalent Aerodynamic Diameter (EAD) into three main fractions: coarse particles of 10 µm or less, referred to as PM_10_, fine particles of 2.5 µm or less, referred to as PM_2.5_, and ultrafine particles of 0.1 µm or less, referred to as PM_0.1_ [3]. 

Historically [4], epidemiologists have documented statistically significant associations between measured airborne PM concentrations in the air and increased morbidity and mortality rates [5,6], as well as health effects related to exposure times [3]. In addition, several physical and chemical properties of fine and ultrafine particles [7,8] have been cited as toxic contributors [9,10] associated with cardiovascular and respiratory diseases [11,12]. It has also been demonstrated that fine particles play a significant role in spreading different viruses such as highly pathogenic avian influenza H5N2 [13], and more recently, their role as a carrier of the SARS-CoV-2 virus has been proposed [14,15]. Other studies have reported that immunosuppression induced by PM_2.5_ exposure could increase the risk of contracting new diseases or reactivating others, such as Pulmonary Tuberculosis, in exposed populations [16].

Despite the tremendous scientific effort to characterize airborne PM, the analysis of its physicochemical composition remains a challenging task due to the lack of a single practical technique to acquire all essential information for the accurate and complete characterization of airborne PM properties, such as their size, shape, individual chemical composition, and mixing state [17,18,19,20,21]. 

The challenge has been the design of a portable, simple, and low-cost device that allows the performance of in situ and real-time airborne PM characterization with good quality control, quality assurance of measurements, and chemometric evaluation data. From this perspective, emerging micro- and nanotechnologies have shown to be a viable option to develop portable analytical systems that can integrate two or more functions.

The integration of microfluidics and micro- or nanoelectromechanical systems (MEMS/NEMS) coupled with signal acquisition devices and data conditioning and processing systems [22,23,24,25] allows multiple functionalities, such as the manipulation, preparation, detection, and analysis, of microsamples in real-time controlled and automated microenvironments with high sensitivity and precision of consumption, leading to a better analytical process throughput [26,27,28] to achieve the Lab-on-a-Chip (LoC)-based point-of-care application [2,23].

A number of technologies have already been proposed and developed to manipulate, detect, and identify airborne PM in gaseous media using LoC devices with high-throughput [29,30] and are reported as an alternative for the classification and environmental monitoring of airborne PM [31,32]. These devices have the ability to separate airborne particles in a number of fractions with target sizes of interest to later provide their corresponding concentration values in the air to determine personal exposure. Moreover, novel microfluidic platforms have been reported for the chemical identification of specific airborne analytes in portable and simple low-cost systems that have proven to serve as prior warning devices with high-throughput and small footprints. However, it is relevant to mention that, although extensively studied, these devices are still in the early stage of development, since very few of them have been taken out of laboratory environments.

Efforts must be made to design robust LoC platforms operating at their full potential with the capacity to perform in situ and real-time classification, mass concentration measurements, and the physicochemical identification of target airborne analytes with a minimum need for human intervention. In addition, these devices can be integrated into real-time atmospheric conditions monitoring stations to transmit information for database generation towards the creation of updated air pollution maps to display the changing properties of airborne PM pollutions to support long-term preventive health strategies that will ultimately reduce airborne PM pollution impacts on public health.

This review summarizes the most significant advances regarding the selected LoC devices found in the literature dedicated to airborne PM characterization. Here, we classified them by their principal applications. Section 2 introduces the principle of operation of microfluidic devices to classify airborne PM by its corresponding fraction. Section 3 introduces the principle of operation of MEMS and NEMS sensors used to detect the airborne PM mass concentration. Section 4 introduces the principle of operation of microfluidic devices employing analytical methods to identify several airborne PM components. 

Very few reviews regarding the subject matter are found in the literature [29,31,32]. The outlook of several authors serves as a research tool to fully understand the full potential of integration to create opportunities towards the development of more robust and novel LoC microdevices for the monitoring, detection, and in situ analyses to provide state-of-the-art research related to airborne PM. 

## 2. Classification Methods

Airborne PM sampling according to their fraction is a critical step in analyzing the air quality, as well as the evaluation of different air pollution sources and the development of epidemiological studies. Many types of high- and low-frequency airborne PM samplers [17,20,33,34], complemented with mass measuring methods [35], and microscopic and spectroscopic analytical systems [36,37,38,39,40,41,42] have been implemented to determine PM morphological and chemical distribution [43,44,45]. Nevertheless, the latter procedure presents a critical issue: the lack of a collection substrate that meets all required instrumental specifications for a single PM sample analysis [19].

In contrast, microfluidic chips allow particle sorting in a continuous flow. These microdevices can be active or passive [46,47,48,49], depending on whether or not an external force is applied to separate particles by one specific property, such as their shape, size, density, or protein components [50,51,52]. Passive technologies’ classification performances depend on two principal characteristics: the geometry of the microchannel, as in Deterministic Lateral Displacement systems and inertial microfluidics [49], and the fluid dynamics, as in hydrophoresis and viscoelastic microfluidics [53,54]. Meanwhile, active technologies allow real-time dynamic manipulation by applying external forces (acoustic, magnetic, optical, electrophoretic, or dielectrophoretic) that induce driving forces to relocate particles to their equilibrium position inside the three-dimensional geometry of the microchannel [49,55,56].

**Figure 1:**
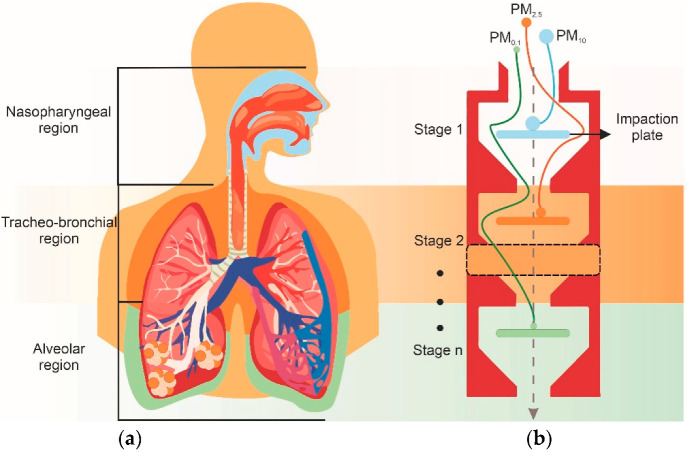
Illustration of (**a**) the predicted fractional deposition of inhaled particles in the nasopharyngeal (blue), tracheobronchial (orange), and alveolar (green) regions of the human respiratory tract during nose breathing, and (**b**) airborne particle classification by the Equivalent Aerodynamic Diameter of PM_10_ (blue), PM_2.5_ (orange), and PM_0.1_ (green) particles using a CI.

Due to their simplicity, passive technologies are among the most frequently applied to separate airborne PM, especially those based on inertial microfluidics. Active-based devices have also been reported. However, their application on airborne PM is less common due to their complexity and continues to be studied [57]. With the growing awareness of health impacts caused by airborne PM exposure [11], most devices have microfabricated inertial impactors, since they resemble particle deposition throughout the different sections of the respiratory system (Figure 1a) and are used to estimate particles’ locations according to their EAD (Figure 1b) [58]. Considering that their working principle is similar to that of inertial microfluidics, inertial impactors are considered a passive technology and will be presented in Section 2.1. Passive and active microdevices for the classification of airborne PM are described below.

### 2.1. Passive Classification Systems

Inertial microfluidic devices use inertial migration to drive particles traveling through a viscous fluid to an equilibrium position according to their density, size, or shape within a system with geometrical symmetry [59,60].

Inertial migration is induced by the sum of an inertial lift force and a drag force, both generated by secondary flows [61,62] produced by the geometry of the microchannel and the intrinsic properties of the fluid. In general, a geometrical design can be simple [63], with straight [64] and curved shapes [65], or of more complex structures with spirals [66] and contraction and expansion arrangements or grooves [54] that, when combined with active technologies, can process multiple types of particles with higher sensitivity [67].

The separation quality of inertial devices is characterized by the Reynolds number (*Re*) in the laminar regime, expressed as:

(1) $$Re=\frac{\rho L_{c}U_{Max}}{\mu }$$

where *ρ* is the density, *U_max_* is the maximum velocity, *µ* is the dynamic viscosity, all from the fluid, and *L_c_* is the hydraulic diameter of the entrance nozzle of the microchannel [68]. 

*Re* associates the fluid effects of inertia and viscosity affecting the radial displacement of particles (Δ) through the streamlines, allowing to control their positions within the microchannel’s geometry [61]. The ability to separate a particle from its original streamline is determined by the separation efficiency (*E*) for curved microchannels [69]:

(2) $$E_{c}=\frac{\Delta }{2w}=\frac{\pi }{2}\theta \left(Stk\right)$$

and for straight microchannels [70]:

(3) $$E_{R}=\frac{\Delta }{w}=\frac{\pi }{2}\left(Stk\right)$$

where *θ* is the bend radius, w is the microchannel’s half-width, and *Stk* is the Stokes number that describes the relationship between the particle stopping distance and the dimensions of the microchannel [70], indicating that if the *Stk* value is above unity, the particle will be collected [71]. The *Stk* is defined as:

(4) $$Stk=\frac{\rho_{p}C_{c}d_{p}^{2}U}{18µW}$$

where *ρ_p_* is the particle density, *d_p_* is the particle diameter, and *C_c_* is the Cunningham correction factor derived from the Stokes law [70]. 

**Figure 2:**
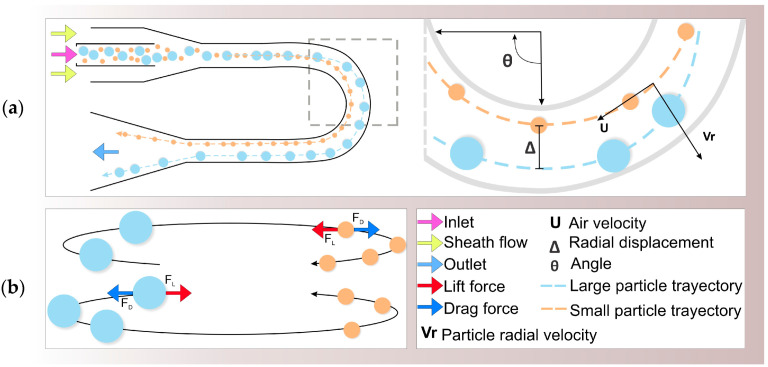
Schematic diagram of the inertial separation principle in a curved microchannel for airborne particle classification. (**a**) Particles mode radially outward in the curved channel due to centrifugal force after their alignment through the application of a sheath flow. (**b**) Dean vortices in a transverse view of the microchannel affecting particle movement.

For particle sorting, inertial classification devices use curved geometries [72,73,74,75]. This type of structure induces a secondary Dean Flow that allows faster particle migration with greater *E* [76]. The Dean Flow effect is generated by centrifugal forces induced by external axial flows that create vortices moving in the opposite direction of the inertial lift force to drag particles to their equilibrium positions [62,77,78], as seen in Figure 2a. Therefore, as long as the magnitude of the drag force does not exceed that of the inertial lift force, particles with similar *EADs* will be focused into single streams, avoiding particle mixing [76]. 

Schaap et al., demonstrated the separation of airborne particles of 0.2 µm and 3.2 µm by centrifugal forces in a novel rectangular U-shaped microchannel [73]. Particles were aerodynamically focused toward the microchannel’s centerline by two sheath air flows to maximize the Δ resolution along a 90° curvature where particles were acted upon by a centrifugal force, according to Equation (2) [72]. As a result, an 80% separation efficiency was achieved [73]. The same effect was also used by Hong et al., for airborne PM, bacteria, and virus separation. The device consisted of two stages with slight variations in their respective sheath airflow rates to control the Dean Flow intensity and particle movement along the microchannels. The experimental results indicated a separation efficiency of more than 65% and a less than 10% particle loss in all three outlets [74].

Both systems showed the reliability of the Dean effect for airborne PM sorting. However, it is necessary to emphasize that particle separation in more than two stages represents a greater challenge, with every stage requiring flow control; therefore, the device’s fluidic and geometrical design becomes complex. 

Xu et al., proposed a system for aerial microorganisms’ separation with two main sections: a particle focusing zone and a particle separation zone [75], i.e., a combination of the operation principles of the previous systems [73,74]. Particles were focused toward the centerline within the first zone by two sheath flows. Later, when focused, particles entering the second zone underwent different degrees of displacement by the influence of a perpendicular drag force generated by a third sheath flow, creating a separation effect similar to the Dean Flow theory, as seen in Figure 2b. The device performance was analyzed using two indicators: separation efficiency and purity between mold spores of 6 µm and 10 µm. The results indicated a separation efficiency of 89% with a purity of 98% [75], demonstrating that sheath flow control improves the separation efficiency for inertial-based classification systems.

#### Inertial Impactors

Impactors are widely studied, since their simple t-form structure allows easier manufacture and higher performance with minimal flow control in comparison with inertial microfluidic devices. Furthermore, impactors can be easily coupled with different MEMS or NEMS sensors (described in Section 3), providing real-time functionalities to monitor and analyze airborne PM classified by specific EADs in several separation stages, characteristics not yet implemented by inertial-based devices [70,71].

**Figure 3:**
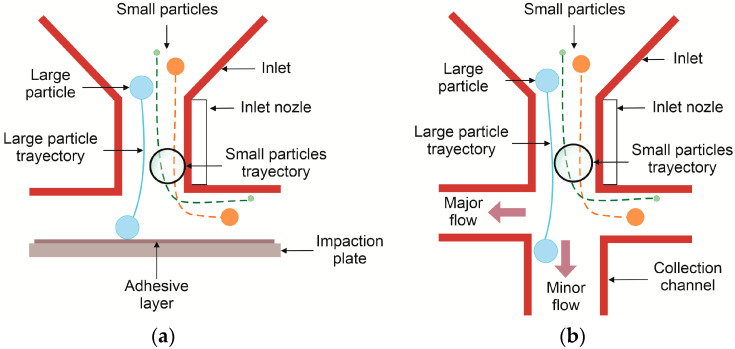
Operation principles of the (**a**) Cascade Impactor (CI) and (**b**) Virtual Impactor (VI).

Impactors have two types of structures: Virtual Impactors (VI) are a modified design of the principle of operation of the Cascade Impactor (CI) composed of a number of stages consisting of a circular or rectangular inlet nozzle and an impaction area to collect aerodynamically classified airborne PM [71,79]. In CIs, a flow entering the nozzle accelerates towards an impaction plate that abruptly changes the direction of the streamlines at a 90° angle such that a centrifugal force, proportional to the particles’ diameter, density, and speed, is applied [70,80]. As a result, particles with an EAD larger than a certain cut-off point with sufficient inertia impact the plate, while smaller particles with less inertia travel along the streamlines to the next stage, as seen in Figure 3a. In a VI, a straight collection channel or virtual region replaces the impaction plate. Consequently, for effective particle classification, the flow distribution inside the VI must be controlled so that 90% of the total flow travels along the lateral or major flow channels, while the remaining flow (10%) follows the collection or minor flow channel direction [70], as seen in Figure 3b.

Unlike inertial microfluidics, the performance of the impactors is measured according to the *Stk* number at which the collection efficiency is 50% (*Stk*_50_) [71,81]. Therefore, if the *Stk*_50_ value is known, the particle’s diameter for which the collection efficiency is 50% (*d*_50_) can be calculated, with this being the cut-off point mentioned above [71]. Assuming ideal conditions for an impactor meeting the design criteria established by Marple and Willeke [81], the optimal *Stk*_50_ values for a rectangular and a circular nozzle are 0.59 and 0.24, respectively [82,83].

(5) $$d_{50}=\sqrt{\frac{\rho_{p}C_{c}Q}{9\mu LW^{2}}}\sqrt{Stk_{50}}$$

**Figure 4:**
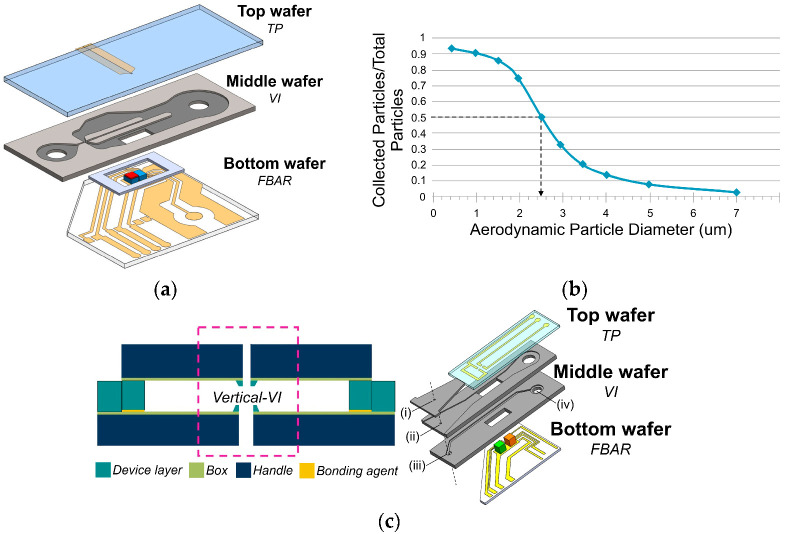
Design of a µIV. (**a**) CAD drawing of a µIV. (**b**) Collection efficiency curve of a μVI obtained by FEM analysis. Modified from Reference [84]. Copyright (2013) with permission from Elsevier. (**c**) Cross-section of a vertically stacked μVI. Modified from Reference [98]. Copyright (2019) with permission from Elsevier.

For the classification of airborne PM applying the *Stk_50_* design criteria, different µVIs and µCIs have been developed. Several research-based devices are discussed and listed in Table A1 in Appendix A. Their differences lie mainly in the number of classification stages and the design *d*_50_ values that rely on the device’s fabrication process, since this will limit their dimensions. In the literature, µ-impactors have a planar physical design similar to that proposed by Paprotny et al., as seen in Figure 4a [84]. These devices can be manufactured through a variety of microfabrication processes [85,86,87,88,89,90,91,92,93,94,95,96,97,98,99,100,101], precise micromachining technologies [84,102,103,104,105], and more recently, 3D printing manufacturing technologies [106,107,108,109,110].

Li et al., designed a micromachined µVI with a rectangular nozzle for PM_2_ classification. The total flow rate (*Q*) was calculated for *Stk*_50_ values from 0.479 to 0.59 according to Equation (5). With an experimental *Q* of 90 mL min^−1^, the µVIs collection efficiency and particle loss at *d*_50_ were quantified at 34% and 33%, respectively. The low collection efficiency was attributed to the differences between the experimental and the calculated *Q* values for a rectangular nozzle, demonstrating the importance to follow the design criteria to obtain a good separation efficiency curve [84].

To improve the μ-impactors design, the air–microfluidics research group, using a Finite Element Method (FEM) analysis, further adjusted the design of a rectangular μVI for PM_2.5_ classification with a low operating flow rate of 6 mL/min, for which an optimal separation efficiency curve was obtained (Figure 4b) [84,88,89]. The same approach was later used by Fahimi et al., to develop a novel µVI-PM_2.5_ device with a vertical geometry that allowed a higher miniaturization level, as seen in Figure 4c. The vertical µVI included an isolated minor flow channel that incorporated an impactor plate to remove coarse particles, reducing the system’s contamination [98].

One major challenge of µ-impactors is particle wall loss (WL) due to particle impaction during its acceleration towards the impaction area. Several particle WL correction methods have been developed and adopted. FEM analysis is commonly used to adapt devices’ design parameters to maintain WLs under a 10% value. Pretreatment configurations have also been developed to improve the separation efficiency and reduce impactors’ WLs. These pretreatment areas are used to focus particles toward the nozzle centerline to create a sharp cut-off curve slope approaching the ideal efficiency curve [99,101].

µ-impactors can also be designed as multistage systems of two [100,102,110], three [85,103], four [111], and up to five separation stages to divide airborne PM into smaller fractions [107]. For multistage devices, their principle of operation is based on that of µCis, since they exhibit a simpler flow control and sharper cut-off curves slope when compared to multistage µVIs [110] in addition to ultrafine airborne particle classification due to a lower pressure drop that lessens the particle fragmentation [87].

For µCis, particle WL can be reduced by coating their impaction areas with oily thin-film layers [103] or by their fabrication with viscous materials [107] to mitigate particle rebound, although this could lead to particle agglomeration. As an example, Kwon et al., designed a five-stage µCI for ultrafine particle classification based on the previous work of a four-stage µCI by Kim et al. [111]. To reduce particle bouncing, the impaction areas were fabricated in PDMS. For each stage at a calculated *Q* of 0.55 LPM, the *d*_50_ values were experimentally determined to be 1.17 µm, 0.94 µm, 0.71 µm, 0.54 µm, and 0.23 µm, where each of them presented a deviation of less than 11% with respect to the theoretical values [107]. Furthermore, the proposed system was designed to monitor ultrafine particles, which cannot be detected by a regular particle size spectrometer.

### 2.2. Active Classification Systems

Active-based particle classification uses external forces to generate driving forces over an incoming flux of particles to relocate them towards an equilibrium position according to a specific property. with separation efficiencies close to those of inertial microdevices. These systems are generally used to separate biological particles from ambient air based on their dielectric constant differences while passing through a non-uniform electric field [83] without limited flow conditions, allowing more accurate separation resolutions between different types of biological and non-biological airborne particles, although passive flow control technologies are commonly integrated for particle positioning [112,113].

**Figure 5:**
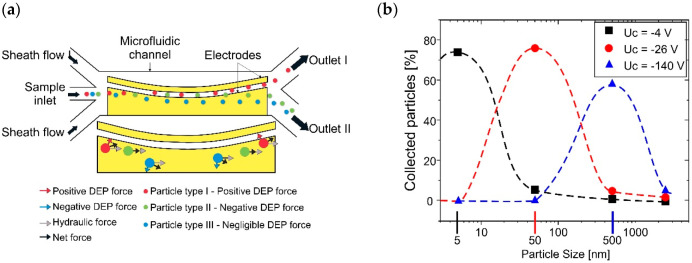
Active separation techniques of selected airborne particles. (**a**) Trajectories of particles under an nDEP force for aerial bacterial isolation. Reprinted with permission from [112]. Copyright (2009) American chemical Society. (**b**) FEM simulation of positive charged NP sampling efficiencie curves. Reproduced from [115]. Copyright (2020) Molecular Diversity Preservation International under a Creative Commons Attribution License available online: https://creativecommons.org/licenses/by/4.0/ (accessed on 14 February 2022).

Moon et al. employed negative dielectrophoresis (nDEP) using a novel curved electrode to achieve rapid separation and isolation of aerial bacteria directly in air. The electrode’s curved shape was intended to attract positively charged bacteria and repel negatively charged dust particles redirecting them towards a specific outlet channel (see Figure 5a). Using this method, bacterial isolation of approximately 90% was achieved [112]. 

Electrophoresis has also been implemented for the real-time separation of biological and non-biological particles of similar size by their electric mobility differences created by a negative corona discharge. Because the number of charges depends on the particles’ relative permittivity (ε) when passing through an electric field, biological particles with a high ε were attracted to a positive-biased electrode while non-biological particles with lower ε moved towards a grounded electrode. Experimental results showed 70% and 80% outlet purity for polystyrene particles and S. epidermidis, respectively [113]. 

A novel study for nanoparticles (NPs) separation by their natural charge is the one carried out by the Peiner research group based on the previous work of Park et al., who proposed the use of micro-cantilevers for NPs monitoring [114]. Bertke et al. theoretically demonstrated the separation and collection of positively charged NPs by their attraction to a micro-cantilever structure with an electric field created by a counter electrode surrounding a negative-biased electrode located on the micro-cantilevers free end surface. Simulations by FEM analysis demonstrated a separation efficiency of 80% for particles of 5 nm, 50 nm, and 500 nm by applying collection voltages of −4 V, −25 V, and −140 V, respectively, as seen in Figure 5b [115]. However, this method is still being studied because experimental results have not been verified [116].

## 3. Detection Methods

Typical methods to measure airborne PM concentrations or total mass exploit gravimetric and optical approaches for a quantitative time-dependent detection to assay the air quality measurements. Although these methods are well-established, there is still an important paradigm between air quality measurements and the real-time personal PM exposure, since both move along with time and space [12]. Personal monitoring devices have become essential in epidemiological studies, because they provide more detailed information about the air quality to which a person is exposed in the short term while moving in their habitual environment. Although these systems present the advantage of being portable, they are also complex, expensive, and bulky [117].

In recent years, MEMS/NEMS sensor technologies have been coupled with the outlet or outlets of microfluidic classification devices for on-field airborne PM detection. These systems usually use electrical or optical stimuli to measure a variable shift generated during particle deposition on a sensing surface or particle crossing in front of a detector. The sensing performance characteristics are expressed in parameters such as their Limit of Detection (LOD), Quality factor (Q_f_), sensitivity, stability, precision, response and recovery times, operation life, and noise level, as well as their manufacturing cost, size, weight, and their ability to be integrated with other devices [118,119]. Their selection depends on the researcher’s technological access, particle size, and method of interest. Table A2 summarizes an overview of sensors applied to airborne particle detection.

### 3.1. Electrical-Based Sensors

An electrical sensor for airborne PM monitoring is based on the measurement of the change in the resonance frequency (resonant sensors), capacitance (capacitive sensors), or current (corona discharge sensors) after particle deposition onto micro- or nano-sensing surfaces. These sensors exhibit a high accuracy and lower power performance in compact packaging as a result of different microfabrication techniques [120]. Their application can be found in the three main PM fractions. Herein, we will briefly describe their working principle and examine several examples applied and designed for airborne PM sensing.

#### 3.1.1. MEMS-Based Sensors

MEMS-based resonance sensors have received particular attention since Sauerbrey established the relationship between the frequency changes of a piezoelectric crystal after a mass load [121]. Environmental scientists have taken advantage of the progress made in the last decades to integrate microgravimetric transducers functionalized for airborne PM detection on scales less than micrograms in devices that allow direct and real-time sensing with high sensitivity. Moreover, these sensors can be easily coupled to μ-impactor structures for direct air quality measurements with resolutions in the nanogram scale. Different acoustic sensing elements exist, varying in operational frequency and acoustic wave type. These devices can be Surface Acoustic Wave (SAW) or Bulk Acoustic Wave (BAW) resonators [122].

**Figure 6:**
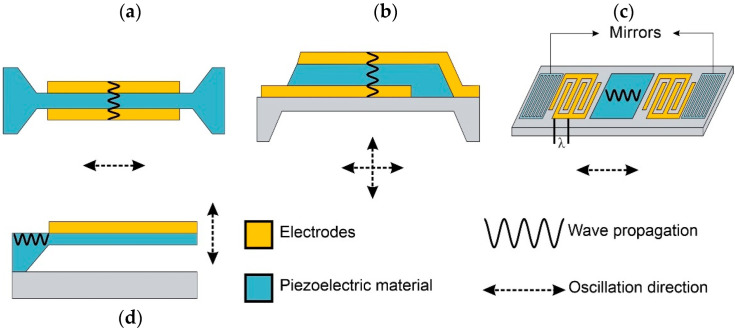
Schematics of electrical sensors. (**a**) Surface Acoustic Wave or SAW. (**b**) Quartz Crystal Microbalance or QCM. (**c**) Film Bulk Acoustic Resonator or FBAR. (**d**) Piezoelectric Cantilever Resonator or PCR.

Quartz Crystal Microbalances (QCMs) and Film Bulk Acoustic Resonators (FBARs) belong to the family of BAW sensors [122]. QCMs are the first generation of acoustic resonators used as gravimetric sensors [121]. A Classic QCM uses the piezoelectric nature of an AT-cut quartz crystal vibrating on its thickness shear mode between two gold electrodes to monitor small changes in the mass, as seen in Figure 6a.

QCMs are the most common and widely used sensors due to their comparatively low fabrication costs in comparison with FBAR and SAW sensors, although the resolution values are limited to typical resonant frequencies in the range of 5–20 MHz, since it depends mainly on the substrate thickness. Moreover, when integrated with μ-impactors, QCMs act as an impactor plate; therefore, the top electrode tends to be covered with adhesive thin films to improve the particle deposition, which can be modified as antigen–antibody-sensing layers to selectively detect airborne viruses [123,124] and allergens [125].

The collection performance can be further improved with heated-QCMs (H-QCM) that increase electrodes’ surface sticking coefficients. Zampetti et al., implemented an H-QCM coated with a grease film resonating at 10 MHz. At 80 °C, the device presented a LOD of 15 µg m^−3^, which was proven to be 2.5 times higher than at room temperature [126].

For QCM sensors coupled with μVIs, particle WL can also play an important role in the diminution of particle deposition. Zhao et al., developed a self-assembled 3D print-based µVI-QCM system for PM_2.5_ monitoring. The QCM sensor with a thin photoresist film coating at a resonant frequency of 4.98 MHz achieved a 142-µg m^−3^ LOD with a mass resolution of 3.47 ng [106]. Later, the same group developed a more compact structure for airborne PM_1_ monitoring. Compared to the previous system, this device presented a higher detection level with a sensitivity of 3467 Hz min^−1^ and a LOD of 52.33 µg m^−3^ due to diminutions in particle WLs in consequence of the microchannel’s length reduction [108].

Although the principle of operation of an FBAR sensor is similar to that of a QCM sensor (piezoelectric film between two metal electrodes), FBARs work at a much higher resonant frequency in the range of 0.5–5 GHz. This characteristic allows higher sensitivities in the sub-nanogram scale (SAW-medium and QCM-low sensitivities) in smaller sensing areas, as seen in Figure 6b, due to a bottom-up fabrication process using thin-film technologies [127]. However, given its manufacturing complexity, the application of FBAR sensors is less common [119].

Particle deposition of FBAR sensors is supported by thermophoresis (TP). Therefore, for airborne PM monitoring applications, μ-heaters are placed above FBAR structures to create a temperature gradient across a microfluidic channel. The air–microfluidics research group was a pioneer in developing airborne PM sensors based on the first report of an FBAR inside a microchannel [128]. Paprotny et al., developed a PM_2.5_ monitoring system integrated with FEM-optimized µVI and a FBAR-TP sensor. With a resonant frequency of 600 MHz, the experimental results suggested a theoretical LOD of 2 µg m^−3^ [84]. Later, Fahimi et al., improved the µVI’s efficiency, with the previously described vertical structure reducing the particle WL. The sensor’s sensitivity was reported at 7 Hz min^−1^ for each µg m^−3^, with a calculated LOD of 1.0 µg m^−3^, which is the highest sensitivity for airborne PM-MEMS sensors reported to this day [98].

In SAW sensors, a Rayleigh acoustic wave induced by an electrical charge generated by an arrangement of Interdigital Transducer electrodes (IDT electrodes) surrounded by reflective grids traveled along a piezoelectric quartz surface, as seen in Figure 6c. In this type of sensor, the intermediate resonant working frequency (100–1500 MHz) can be varied, adapting the acoustic wave penetration depth (*λ*) to a specific particle size, allowing acoustic coupling of the entire particle volume of interest, with resolutions close to those of an FBAR device [119].

To enhance the performance, SAW devices are fabricated in more compact designs that allow higher operational resonant frequencies. Thomas et al., developed a novel SAW sensor based on a thin-film Solidly Mounted Resonator operating at 894 MHz. The tests indicated a more stable response to fine particle measurements with a higher sensitivity of 7.5 kHz for each µg m^−3^ [129] when compared to the previous design [130].

SAW devices have also been implemented in µVI structures for fine particle measurements. Liu et al., integrated a SAW sensor [131] in a µVI-TP device, both improved by the FEM analysis. The sensor’s performance was analyzed using monodisperse polystyrene latex (PSL) particles for which a LOD of 2 µg m^−3^ and a mass resolution of 0.17 ng were obtained [96]. Adhesive films have also been implemented in µVI-SAW devices to enhance particle deposition. A glycerol film-coated SAW sensor resonating at 147.24 MHz reached a linear sensitivity of 7.46 Hz min^−1^ for each µg m^−3^, around 133.4 times higher when compared to the µVI-QCM device, making it more suitable for fine particle detection [109].

#### 3.1.2. NEMS-Based Resonance Sensors

Although classified as BAW sensors, NEMS resonators are introduced separately due to their higher sensitivity and resolution, making them suitable for detecting the mass of individual NPs that have not been carried out with SAW, QCM, or FBAR sensors [132]. Thermal NEMS Resonators (TPRs) have successfully been integrated in µCIs, working at high resonant frequencies and reaching Q_f_ values of 20,000 with resolutions up to 2.3 pg [133] and mass sensitivities as high as 1.6 kHz/pg [134], sufficient to detect individual NPs. However, due to their tendency to remain airborne in consequence of Brownian motion [135], NP deposition occurred under partial vacuum to improve the sampling efficiency, resulting in integration times of several hours [133,134,136]. NP sampling has also been achieved by their inertial collection at high aerosol velocities into nanomechanical resonant filter fibers with an efficiency of around 65% [135]. Nevertheless, high aerosol velocities can lead to particle fragmentation at the moment of impact [87].

nDEP attraction has also been used to improve NP attraction onto the surface of Piezoresistive Cantilever-based Resonant sensors (PCR). These sensors consist of a negative electrode placed at the bottom of a PCR structure to generate an electrostatic field to attract positively charged NPs, as seen in Figure 6d, allowing particle sampling in the air with times of several minutes [135]. After NP sampling, a PCR that resonates between units of hertz to a few megahertz in its fundamental resonant mode with 10^2^ to 10^3^ Q_f_ values lowers its resonant frequency. The frequency shift is detected by a full Wheatstone bridge [135,137] that replaces the conventional optical detection method, increasing their portability [135,138,139]. This system configuration achieves resolutions up to 1 pg with high sensitivities [137,140,141]. For further enhancement of the mass sensing resolution, the use of higher resonant modes to increase PCRs’ Q_f_ values has proven to be particularly more effective over reducing their dimensions [142,143,144].

The Peiner research group carried out a series of works describing the design of a novel portable airborne NP-PCR detector or CANTOR. The device was fabricated according to the resonator Q_f_ modification to achieve higher resonant frequency values [143,144]. The CANTOR was later improved, including a microfilter and an impactor plate, to remove coarse and fine particles, followed by a thermal-PCR sensor, a tracker, electronic circuits, and an LCD screen. The CANTOR-2 achieved a calculated LOD of 5.0 µg m^−3^ at a response time of about 6 s [145]. Additionally, for this device, the measurement precision was reported at less than 14%.

The same group also investigated the use of nanopillars to improve PCR sensors’ sensitivity. For this, Wasisto et al., studied NP sampling enhancement using a silicon nanopillars array with an individual resonant frequency of 452.33 kHz [146]. The high-frequency value allowed the generation of a higher electric field region with a higher collection efficiency that increased the detector mass sensitivity by approximately 0.7 million times compared to previously designed PCR sensors [146]. Later, the nanopillars principle was used to improve the cantilever’s NPs sampling. With this arrangement, the cantilever collection efficiency increased to 1.5 times greater than the cantilever sampling efficiency without nanopillars [147]. Forthcoming, their integration within the CANTOR will be the next step to improve its sensitivity, mass resolution, and response time [145].

Additionally, cantilever structures were proven to be reusable by going through different wet and dry washing processes, like an ultrasonic acetone bath, to remove a previously deposited photoresistant film coating the surface of the cantilever [148,149], a nitrogen gas purging method [146], and a polydimethylsiloxane (PDMS) deposition and removal process [146].

#### 3.1.3. Capacitive-Based Sensors

**Figure 7:**
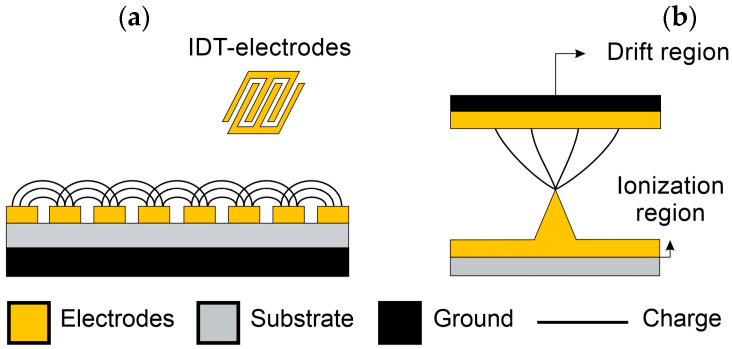
Schematics of electrical sensors. (**a**) Capacitive. (**b**) Corona discharge.

Capacitive sensors are based on impedance variations due to particle/electric field interactions occurring while a single particle precipitates towards an electrode surface, increasing its capacitance (Figure 7a). These sensors allow a coarse and fine particle granulometry analysis, i.e., size distribution analysis, which is highly relevant from a toxicological perspective and cannot be determined by other MEMS/NEMS sensors due to particle agglomeration [150]. Glass coplanar configurations integrated with IDT electrodes have been applied in the detection of single airborne PM_10_, with the experimental results validated by optical and FEM analysis, demonstrating a good correlation between the capacitance variations and particle volume with resolutions of about 1.2 aF [150]. Furthermore, fine particle measurements have been carried out by high-resolution capacitive sensors with an IDT microelectrode architecture lock in a CMOS chip that reduced the parasitic effect, improving the sensor’s resolution to an average of 65 zF, for which particle detection of less than 1 µm becomes achievable [151]. TP-based particle deposition has also been integrated with sampling enhancements up to 84% in devices with a capacitance sensitivity of about −56.8 pF µg^−1^ [93].

Recently, Oluwasanya et al., designed a novel microfluidic module for PM monitoring based on particle motion under TP influences to separate particles into different streams, depending on their size. For particle detection, coplanar IDT electrodes were arranged according to the trajectory of the PM_10_ and PM_2.5_ streams. FEM analysis showed a capacitive sensitivity of 0.48 zF per fine particle. However, this method continues to be studied, given the necessity to integrate environmental and electric parameters that may reduce the sensor’s sensitivity [152].

#### 3.1.4. Corona Discharge Sensors

Corona discharge-based MEMS sensors have proven to perform ultrafine particle concentration measurements [153,154]. In addition, these sensors offer the advantage of being easily integrated into microchannels for real-time in-flow detection, unlike previously described sensors in which particle deposition is necessary [86,155]. A corona discharge (CD) sensor consists of two main elements: an ionization region, composed of a silicon tip electrode, above a drift region (Figure 7b). When passing throughout the drift region, particles are charged with positive ions discharged from the ionization region. Then, particle concentration is determined by measuring the particle electrical current [154] with sensitivities comparable to commercial instruments [107,156].

Kim et al., developed a µVI-CD system for ultrafine particle classification and detection. Ti-Cu electrodes carried out particle number concentration measurements with a sensitivity value of 8 × 10^−7^ pA (# cm^−3^)^−1^ [86], with experimental results equivalent to those of a condensation particle counter [156]. Later, for more accurate concentration measurements, a particle precipitation section and a detection section were coupled between the µVI and CD structures. First, a precipitation electric field was applied to determine the particles’ mean diameter in the precipitation section. Then, the particles were collected in a metallic filter for concentration measurements. The experimental results showed a calculated concentration range from 320 to 10^6^ cm^−3^ for ultrafine NaCl particles [95].

However, for real environmental applications, narrower particle fractions are necessary to provide more accurate information regarding their concentration. For this purpose, Kwon et al., developed a five-stage µCI (described in Section 2) integrated with a µCD sensor. Ultrafine particles were charged, classified, and collected onto current-sensing impaction Cu-electrode surfaces. The experimental measurements for polydisperse titanium oxide particles ranging from 0.11 µm to 1 µm presented a standard deviation between 11.2% and 6.3% from a commercial aerodynamic particle meter [107].

### 3.2. Optical Sensors

Optical sensors are the most widely used in low-cost PM_10_ and PM_2.5_ portable air quality meters. These sensors measure light scattered by particles as they pass through a beam of monochromatic light. In the literature, different modules integrated with optical sensors and inertial classifiers, including those based on microfluidic technologies [91,92,157] that have also been integrated into mobile applications [105] and even applied to NP detection [158], have been reported. However, these systems are still considered bulky due to packaged optical elements.

The Zhang research group developed a microfabricated camera with two mounted micromachined silicon components with an infrared laser diode and a photodiode to reduce the optical sensor dimensions. The preliminary results showed a calculated LOD of 32.8 µg m^−3^ for a smoke sample of 300 µg m^−3^ [159]. Later, the same group developed a single micromachined silicon chip composed of a two-paired light source and photodiode arrangement mounted in a microfluidic flow chamber. The experimental results indicated a sensitivity of 10 µg m^−3^ [160]. Subsequently, the microfluidic flow chamber was replaced by a µVI structure for PM_2.5_ classification and detection, with which a sensitivity of 2.55 µg m^−3^ was obtained [94].

## 4. Analytical Methods

In the air pollution science field, microfluidic devices provide platforms capable of facilitating airborne PM sampling, detection, and bioassays. Numerous papers in the literature describe microdevices with principles of operation based on continuous flow microfluidics [161,162], droplet microfluidics [29], or paper microfluidics [163,164]. These approaches use specific reagents to identify specific atmospheric components, such as metallic ions, volatile organic compounds (VOCs), and airborne pathogens, in addition to the analysis of the oxidative aerosol response of airborne PM samples with colorimetric [162,163,164,165,166,167,168,169,170,171,172,173,174,175,176], electrochemical [177,178,179,180,181,182,183,184,185,186,187], optical [188,189,190,191,192,193,194,195,196,197,198], or spectroscopic assays [199,200,201]. For previous analytical approaches, their principles of operation, target airborne analytes, and LODs are summarized in Table A3. The following sections will describe different LoC devices intended for airborne PM identification.

### 4.1. Continuous Flow Microfluidics

Continuous flow microfluidic devices allow manipulating a constant flow through a microchannels arrangement using MEMS-scale external pumps. These devices use particle-into-liquid samplers (PILS) [182], gas–liquid interfaces [180], or sampling microchannels washing [189] to mix previously collected airborne particles into reactive or colloidal suspensions to obtain an electric or an optical response based on the target of the assay.

#### 4.1.1. Electrochemical-Based Detection

Microelectrode arrays have been incorporated into microfluidic devices for rapid electrochemical analysis with minimal performance loss, low fabrication costs, and ease of implementation and disposability compared to traditional electrochemical sensing devices. Furthermore, measurements can be universal via conductivity detection [180], semi-selective via amperometric detection [182], and highly selective when combined with capillary electrophoresis [178,179] or by the electrode’s surface modification [181,186].

Electrochemical detection has an advantage over colorimetric and optical-based detection, since the multiplex detection of trace levels of a variety of aerosol constituents has been reported with high-throughput. Noblitt et al. [178] and Dossi et al. [179] used capillary electrophoresis (CE) for the simultaneous electrochemical determination of trace levels of common atmospheric constituents. The conductive detection of a mixture of sulfate anions (SO_x_), nitrate (NO_3_), chloride (Cl), and oxalate (C_2_O_4_) was achieved by dissolving the sample in dilute background electrolytes (BGE) to improve the separation resolution and sensitivity, with limits of detection below 250 nM [178]. Moreover, the presence of aliphatic aldehydes in environmental samples collected with 2,4-dinitrophenylhydrazine (DNPH) cartridges were derivatized to form DNPH hydrazones later eluted into an electrochemical system. Experiments to detect formaldehyde, acetaldehyde, and 2-propenal showed LODs with values of around 10 µM [179].

**Figure 8:**
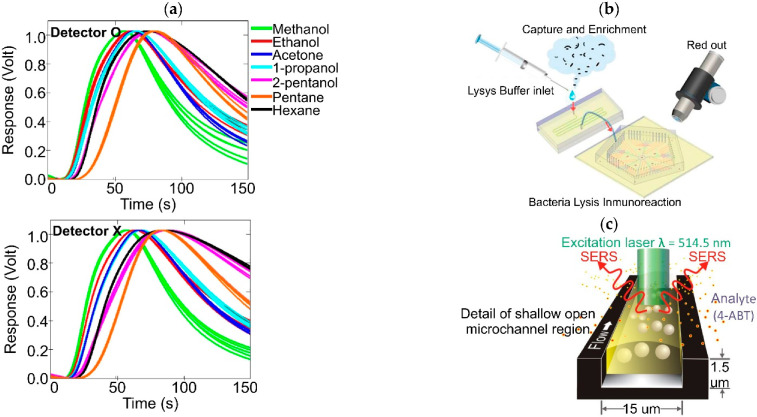
Continuous flow microfluidic techniques for the detection of PM in the air. (**a**) Responses of the O detector and the X detector to different gaseous analytes. Modified from Reference [185]. Copyright (2017) with permission from Elsevier. (**b**) Schematic diagram of a LAMP-based microfluidic device for airborne bacterial identification. Reprinted with permission from Reference [190]. Copyright (2014) American chemical Society. (**c**) Schematic diagram microfluidic/SERS analytical device. Modified from Reference [199]. Copyright (2007) National Academy of Sciences of the USA.

Paknahad and Hoorfar developed a novel VOC identification technology based on the selective sensing capacity of a commercial chemoresistive gas sensor studying the effects of different metallic-coated microchannels on gas molecules diffusion–physisorption [185,186]. Two microfluidic channels with different inner coating combinations were fabricated: a microchannel coated with gold, chromium, and Parylene C or O detector and a microchannel coated with gold, chromium, Parylene C, and Cytonix or X detector. The two inner surface coatings, made out of a mixture of film-forming materials, were used to analyze the conductive response of the sensor to alcohols, ketones, and alkanes. The experimental results showed a higher diffusion rate with the X detector when compared to O detector measurements due to the microchannel’s wall coating lower polarity. This property significantly altered the position of the characteristic vector for each analyte, offering higher selectivity against polar and nonpolar gases, as seen in Figure 8a [186].

Sameenoi et al., analyzed the urban oxidative activity of industrial PM samples through the oxidation of dithiothreitol (DTT) in a PILS sampler. DTT reduction was measured by cyclic voltammetry using cobalt (II)-phthalocyanine-modified carbon paste electrodes (CoPC-CPE) with good selectivity for the catalytic oxidation of organic compounds. The online system presented a detection range from 7 ng to 214 ng, with consumption rates corresponding to analyte concentrations [182].

The detection of aerial pathogens has also been reported. Shen et al., developed an online system consisting of a bioaerosol-in-hydrosol electrostatic sampler and an integrated microfluidic chip with selective antibody-modified silicon nanowire transistors (SiNW-FET) for the detection of the H3N2 airborne influenza virus. Conductance measurements were made to analyze virus concentrations in different air samples. The experimental results revealed higher detection levels for the online microsystem when compared to the quantitative Polymerase Chain Reaction (qPCR) detection limits, with concentration measurements lower than 10^4^ viruses L^−1^ [181].

#### 4.1.2. Optical-Based Detection

Optical detection methods are frequently used for the biological identification of aerial bacteria throughout the measurement of the fluorescence or bioluminescence generated by their exposure to specific colorimetric reagents. An airborne sample is first collected and enriched in a microfluidic chamber to be later washed by a lysis buffer into an immunoassay-based microfluidic chip for its optical identification [189,191]. This process has proven to be a faster and more efficient assay when compared to conventional laboratory culture detection methods [191] or molecular detection using qPCR [188] and Enzyme-Linked Immunosorbent Assay or ELISA [190].

Furthermore, optical-based microdevices have shown the ability to identify target airborne pathogens in times that range from a few minutes [188] to a few hours with higher detection rates, demonstrating their potential use for prior airborne disease warning. As an example, an optofluidic system for influenza A H1N1/2009 virus detection in aerosols from human coughing through a latex immunoagglutination assay was developed. In addition, intensity measurements were detected with a cellphone camera with LODs under 10 pg mL^−1^, increasing its potential of portability [188].

Multiplexed optofluidic devices have also been developed. A Fudan University research group published a series of works describing two novel approaches to identify frequently found airborne bacteria using bacteriological immunoassay analysis (IA) [190] and loop-mediated isothermal amplification (LAMP), both integrated by a capture and enrichment microchamber [189], and a continuous flow microfluidic chip, as seen in Figure 8b [191,193]. With this assembly, a fluorometric-based IA reaction using Ag85B antigens to identify M. tuberculosis with a minimum concentration detection level lower than 10^2^ cells mL^−1^ was achieved [190]. Subsequently, the system was redesigned to identify *S. aureus*, *E. coli*, *P. aeruginosa*, *C. koseri*, and *K. pneumoniae* employing LAMP analysis, which, to their knowledge, was the first report of this type of on-chip detection. The system sensitivity was approximately 24 cells per reaction using *S. aureus* as the representative bacteria [191]. Both systems were used as the basis to design the first portable direct LAMP analysis device for airborne pathogens using a disposable microfluidic chip incorporated into an optical detection module [193], opening up opportunities to develop affordable technologies with the capacity to perform laboratory-based analytical processes with high-throughput.

Optofluidic systems have also been used to distinguish between biological and nonbiological aerosols by differences in their fluorescence intensities, in which detection efficiency measurements are more accurate when compared to traditional methods [197]. These systems use fluorometric intensity measurements by image processing software to measure the concentrations of biological aerosols when in contact with coloring mediums. The detection of B. subtilis, *E. coli*, and *S. epidermidis* dyed with SYTO82 [197] and SYBR green I [90] and by their ATP extraction has been implemented [198]. Linear growths between the bacterial concentration and signal intensity were observed [197,198], demonstrating a superior performance to colony cell counting, with normalized cell concentrations of approximately 88% and 73%, respectively [197]. Moreover, this approach has been integrated into μ-impactors to identify target bioaerosols by their EADs directly in the air. Bioaerosol staining was made during their impact on an agar plate coated with dye [90].

#### 4.1.3. Spectroscopy-Based Detection

Microfluidic devices have been integrated with different spectroscopic platforms to enhance the procedure’s efficiency by providing submicron spatial resolution with high sensitivity and selectivity for different cells and particles [202,203,204]. However, for airborne PM and atmospheric gases, their application continues to be a research area under development.

Piorek et al., described two novel approaches for airborne and explosive molecule sampling and detection based on gas–liquid interfaces with silver nanoparticle (AgNP) colloidal suspension to form “hot spots” for Enhanced Surface Raman Spectroscopy (SERS) analysis, as presented in Figure 8c. First, the induced aggregation of gaseous 4-aminobenzenethiol (4-ABT) was performed to analyze hot molecule formation throughout the measurements of SERS intensities [199]. Then, a continuous sampling system was used to increase the concentration of vapor trace analytes. The detection sensitivity was demonstrated using 2,4-dinitrotoluene (2,4-DNT) vapor at one ppb. Intensity amplification at SERS hot spots up to 10^10^ with a signal-to-noise ratio of 20:1 and an integration time of 2 min was obtained [200]. To enhance SERS signal amplification, SERS-hot NPs clusters were generated throughout a segmented flow, increasing the analyte concentration due to active mixing. Experiments with 4-ABT vapor demonstrated a resulting concentration by a factor of approximately two orders of magnitude higher, reducing the integration time to 2.5 s per spectrum [201].

### 4.2. Droplet Microfluidics

Unlike in continuous flow-based microfluidics, where collected particles are transported directly by a constant flow, in droplet microfluidics, discrete microdroplets carrying collected particles are moved along by a continuous and less dense flow. The generation of microdroplets is based on the interfacial tension between two immiscible liquid phases, like water-in-oil. These microdroplets work as microreactors where collected particles react directly with a specific colorimetric reagent, facilitating faster analyte/assay reactions with a small footprint [205]. Furthermore, electrowetting technologies can be coupled for parallel automatization, facilitating microdroplet handling [206,207,208].

#### Colorimetric-Based Detection

Tirandazi et al., developed a droplet microfluidic device to collect gaseous analytes inside water microdroplets through a high-speed airflow approach method into an oily medium to facilitate their transport along the microchannels. The system’s performance was analyzed by detecting different levels of NH_3_. The samples were captured in Nessler’s reagent microdroplets, resulting in their colorimetric change with an intensity corresponding to the precipitate generated within the microdroplets [184].

Microdroplet devices can also be directly coupled to environmental samplers for atmospheric particle analysis. Damit used a fine aerosol aerodynamic focusing sampler to capture particles in an air–liquid interface through a T-junction for microdroplet formation. The distinction between *E. coli* and non-aerosols was achieved through the real-time microscopic observation of the fluorescent profile of *E. coli* produced with the propidium iodide (PI) microdroplet assay [194].

These platforms have also been used to study atmospheric ice-nucleating particles (INPs). Tarn et al., analyzed atmospheric INPs extracted from a membrane filter and transported them to a microfluidic chip with a cold Peltier-type stage for microdroplet generation, homogeneous freezing, and collection. When frozen, INPs change from a light color to a dark color, facilitating their distinction by time differences in which the freezing events occurred. The experimental results showed freezing events for P. siringae and K-feldspar mineral dust at approximately −3.8 °C and −17.2 °C, respectively [195]. The device was improved by adding a continuous flow freezing channel for the real-time observation of INP freezing phenomena [196].

**Figure 9:**
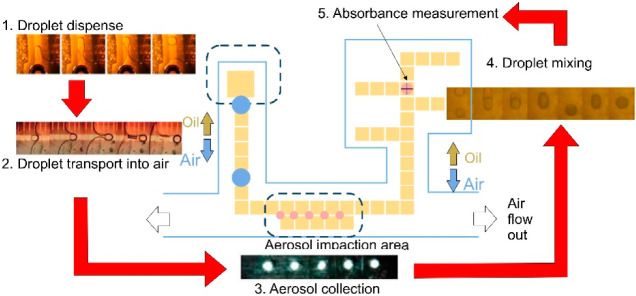
Schematic diagram of an electrowetting device for airborne PM analysis. Reproduced from Reference [166]. Copyright (2020) Molecular Diversity Preservation International under a Creative Commons Attribution License available online: https://creativecommons.org/licenses/by/4.0/ (accessed on 14 February 2022).

One of the main drawbacks of the previously described techniques is the necessity to mix reagents into the aqueous medium before microdroplet generation, which makes them susceptible to large amounts of reagents and human errors. Electrowetting technologies, also known as digital microfluidics, allow microdroplet handling within a microfluidic chip through an arrangement of microelectrodes that function as a microdroplet transport band structure [206,207,208]. With this, microdroplets can be transported, stored, mixed, or analyzed using a set of basic instructions without the necessity of external pressure sources. Fair et al., developed a novel microfluidic device combining an impactor onto the surface of a digital microfluidic chip [165]. Based on this design, Huang et al., applied the same approach for the detection of SO_4_, NO_3_, and NH_4_ ions (see Figure 9). The SO_4_ and NH_4_ experimental results showed LODs of 11 ppm and 0.256 ppm, respectively. However, with the NO_3_ samples, it was impossible to get stable readings due to the polluting precipitate generated inside the microdroplet by the coating surface damage of the mixing area produced by the NO_3_ assay acidic reagent. Therefore, it is necessary to consider the characteristics of the reagents that may affect the electrodes’ surface [166].

### 4.3. Paper Microfluidics

Paper microfluidics have played a significant role in multiplexed point-of-care chemical analysis since their introduction by the Whitesides research group when Martínez et al., presented the first paper-based analytical device or µPAD [209]. These devices have mechanical properties that include simplicity, flexibility, and lightness, with manufacturing prices of around $0.05 per unit for a wide range of clinical and environmental applications, allowing on-site diagnostics, since these devices do not require external instruments to operate, unlike previously described traditional microfluidic devices.

Although there is a great variety of literature referred to as paper microfluidics, this work only focuses on those applied to the analysis of airborne analytes that fall within the framework of the Whitesides research group, where the fluid flow moving along a paper-based chip is driven by capillary forces and controlled by the porosity and geometry of the microchannels. However, we encourage readers to investigate the use of paper as a detection method for a wide variety of airborne analytes in more detail.

Typically, paper-based microfluidic devices are used for the detection of airborne metals, such as iron (Fe), copper (Cu), nickel (Ni), chromium (Cr), magnesium (Mn), lead (Pb), and cadmium (Cd) using the metal–ligand complex principle. Additionally, measurements of the oxidative potential of airborne PM samples through DTT oxidation, with results comparable to traditional electrochemical methodologies, have been reported [170,182,210]. These systems are manufactured by wax microchannels printed on Whatman graded filter paper that is later exposed to a heat treatment to create hydrophobic barriers for flow control. Airborne PM is collected with filter-based samplers to be later neutralized by acidic digestion of solubilize metals into the paper-based chip to proceed with the colorimetric or electrochemical analysis with detection limits of tens to hundreds of parts per million [211].

#### 4.3.1. Colorimetric-Based Detection

The Volckens research group was the first to apply multiplexed colorimetric detection in paper-based chips for concentration measurements of Fe, Cu, and Ni in ash samples from medical residues with LODs of less than 1.5 µg and a linear range from 1 µg to 17 µg for each analyte [167]. The detection of the total Cr in airborne PM has also been reported with a LOD of 0.12 µg and a linear range from 0.23 µg to 3.75 µg [169]. Metal concentrations were quantified by measuring the color intensity of pre-scanned μPADs with image processing software. Metal concentrations were quantified by measuring the color intensity of pre-scanned uPADs with image processing software.

**Figure 10:**
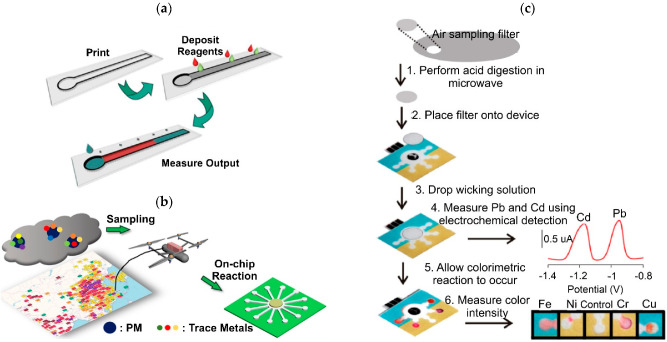
Paper-based microfluidic techniques for airborne PM detection. (**a**) Schematic diagram of a distance-based µPAD. Reprinted with permission from Reference [211]. Copyright (2015) American chemical Society. (**b**) Schematic diagram of a UAV-based approach for airborne particle sampling. Reprinted with permission from Reference [175]. Copyright (2019) American chemical Society. (**c**) Principle of operation of a mPAD. Reprinted from Reference [183] with permission from AIP Publishing.

To enhance paper-based colorimetric detection first, a pretreatment zone and stabilizing agents were added to integrate metal digestion and give the device a long-term shelf life [169]. Following this, masking agents were added into the pretreatment zone to reduce the interference from nontarget analytes [173]. The external-based detection, i.e., scanned image and processing image software, was replaced by distance-based detection where the colorimetric reaction occurred along a capillary path with measuring lines, making naked eye measurements possible, as seen in Figure 10a [173,211]. With this, the uPAD sensitivities were greatly improved by about 50% [173] from previous reports [167,168,172].

Colorimetric detection has the advantage of being easily coupled to portable smart devices, allowing on-site analyses [174]. Jia et al., developed the first paper microfluidic platform based on airborne PM sampling by an unmanned aerial vehicle (UAV) and colorimetric detection through a cell phone application with higher sensitivities when compared to the Volckens group methodology. [174]. The previous system was later paired with a portable reaction kit [176] and a self-built UAV mounting sampler achieving the space-time mapping of airborne polluting metals, this being the first demonstration of the technique (see Figure 10b). With this assembled, the multiplexed quantification of Co, Cu, Fe, Mn, Cr, and Ni was reported at the nanogram scale [175]. To upgrade the system’s sensitivity, uPADs were coated with a graphene oxide nanosheet working as a color-intensifying agent. The results showed an increase in the LOD for Fe by about 2.5 orders of magnitude and for Cu and Ni by a factor of about ten orders of magnitude compared with previously reported measurements [177].

#### 4.3.2. Electrochemical-Based Detection

Paper-based electrochemical detection devices, or ePADs, are attractive due to their high sensitivity, selectivity, and direct measurements with LODs at the nanogram scale compared to standard colorimetric detection. For ePADs, stripping voltammetry with modified bismuth electrodes is the method of choice for the quantitative determination of the trace metal levels. In the environmental area, Nie et al. [212] developed the first ePAD device to detect Pb and Zn in wastewater samples based on the first report of an ePAD by Dungchai et al. [213].

For airborne PM identification, Rattanarat et al., developed a combined multilayer µPAD/ePAD system or mPAD. The multilayer structure was intended to allow multiple measurements with high-throughput, allowing the detection of extremely low trace airborne metals. For the mPAD, the colorimetric layer was based on the previously described methodology for Ni, Fe, Cr, and Cu detection [172,183], while the electrochemical approach was used to determine the concentration levels of Cd and Pb (see Figure 10c). The colorimetric LODs were found below 0.75 µg, while the electrochemical LODs were below 0.25 ng, demonstrating a greater sensitivity against the colorimetric methodology [183].

More recently, Mettakoonpitak et al. [187] developed the Janus ePAD based on a previous work where modified carbon electrodes (Nafion/BiCSPE) were proven to enhance the LODs for Co and Ni in aerosol samples up to 15 times in magnitude compared to measurements by a regular CSPE electrode [214]. The Janus ePAD was developed to simultaneously detect Cd, Pb, Cu, Fe, and Ni in airborne PM samples, achieving LOD values below 0.2 µg for each analyte. The measurements were validated by their comparison with mass spectrometry [187].

## 5. Future Perspectives

In the field of airborne pollution sciences, LoC devices have demonstrated the ability to perform airborne PM monitoring and detection using different operating principles with simple and low-cost devices with fast response times and the capacity to become portable. These advantages allow real-time air quality analysis in addition to the mass concentration determination of some metallic ions, VOCs, pathogens, and even NPs, whose presence in the air represents an environmental health risk. Although LoC devices for airborne PM monitoring are well-established, the analytical methods are still in the early stage of development, since they involve additional complexity by combining several processes. In a perfect world, these microdevices would allow multiple airborne PM identifications on-site. However, their analysis is made after their collection using specific reagents to identify specific airborne components, prolonging the atmospheric pollution characterization process, in addition to the considerable mixing of state variations due to sample resuspension [215].

The mixing state of airborne particles is the main factor affecting airborne PM identification. For most airborne particulates, their total toxicological capacity has not yet been fully understood because of their complex properties, since the internal and external physicochemical structures of every airborne particle can change unpredictably in time and space according to environmental fluctuations [9].

Different analytical techniques are discussed in the literature as alternatives to overcome this barrier. The spectroscopic analysis of individual airborne PM by optical tweezers under controlled laboratory conditions has been reported [216,217,218,219,220,221]. These types of traps have also been incorporated within microchannels for the online and real-time analysis of different types of cells [222,223,224], bacteria [225], and microdroplets [226] using vibrational spectroscopic techniques, of which Raman spectroscopy has proven to be the most compatible with microfluidic platforms [202]. However, as a point of reference to what was considered for this work, there is still no report of the development of microfluidic optical traps for the direct spectroscopic analysis of airborne PM.

In summary, in the last ten years, LoC technologies have proven to be a reliable option for their implementation in air pollution sciences. Air quality monitoring is now possible with portable microdevices to carry out the collection, classification, and mass measurements of airborne PM samples. Research on microfluidics applications in identifying specific airborne PM components has recently shown remarkable progress. However, some of these technologies cannot be taken out of the laboratory environment due to the reading devices being bulky and dependent on human manipulation.

Although LoC devices for air quality monitoring have not yet replaced well-established environmental monitoring techniques, the recent and ongoing research and development have proven to serve as complementary technologies to the current air quality monitoring methodologies. There is still a wide range of opportunities for overcoming technological barriers concerning the evolution of more robust airborne PM analytical microdevices. Efforts must be made to develop portable LoC platforms, since there is still a lack of forward real-time analyses in which, although the portability of these devices has been reported, technologies are separated and must be integrated to achieve the complete characterization of airborne PM. Using novel MEMS/NEMS technologies to integrate and provide continuous data of multiple variables considering airborne PM collection, classification, measurement, and analysis will allow us to improve the state-of-the-art tests regarding the extremely complex nature of air pollution that, to this day, continues to be a scientific and technological challenge.

## Appendix A

**Table A1:** Comparison between the key design and performance parameters of μ-impactors found in the literature.

Type		Fabrication Method	Width W (μm)	*Stk*_50_	Flow Rate *Q* (mL/min)	Cut-Off Point *d*_50_ (μm)	Experimental *d*_50_ at 50% (μm)	Sensing Device	Ref.
µVI	1	a: DRIE on Si Wafer	–	0.59	6	2.5	–	FBAR	[84,88,89,97]
	1	a: DRIE on Si Wafer	190	0.24	9.5	2.5	d_50_ at 45%	FBAR	[98]
	1	a: DRIE on Si Wafer	200	0.59	6.5	2.5	–	Optical	[94]
	1	a: Patterned DFP	1000	0.479–0.59	300	2.5	–	Optical	[92]
	1	a: Patterned DFP	1000	0.479–0.59	300	2.5	–	Capacitive	[93]
	1	a: Patterned DFP	210	0.229	500	0.3	330	Corona discharge	[95]
	1	a: Patterned SU8	200	0.229	300	Type I—0.6 Type II—1.0	Type I—550 Type II—1.1	Corona discharge	[86]
	1	a: Patterned SU8	200	0.229	300	1.0	0.95	–	[87]
	1	a: DRIE on Si Wafer	–	0.59	5	2.5	–	–	[99]
	1	a: Molded PDMS	290	0.372	93.5	2.5	1.93	–	[101]
	1	a: ICPE on Si Wafer	290	0.55	12.5	2.5	–	SAW	[96]
	1	c	1000	0.229	440	1	1.05	SAW	[109]
	1	c	1200	0.59	480	2.5	–	QCM	[108]
	1	c	1000	0.58	270	2.5	2.65	QCM	[106]
	1	b	500	0.479–0.59	90	2.0	*d*_50_ at 34%	–	[104]
	1	b	1000	0.479–0.59	750	2.0	–	Optical	[105]
	2	b	1800	0.23	1000–900	3.15–2.5	3.2–2.28	QCM	[102]
	2	d	500–200	0.59	6.9	10–2.5	–	–	[110]
	3	a: Patterned SU8	3700–1850–350	0.229	600–270–3	0.2–2.5–6	0.135–1.9–4.8	–	[85]
µCI	1	a: Molded PDMS	–	–	500	2.5	–	Optical	[91]
	2	a: Molded PDMS	500–140	0.4–0.8	12.5	5–1	4.83–0.98	–	[100]
	3	a: Molded PDMS	1287–472–263	–	120	2.02–0.88–0.54	2.24–0.91–0.49	Optical	[90]
	3	b	374–197–110	0.72	500	1.06–0.55–0.26	1.19–0.51–0.27	–	[103]
	4	a: Molded PDMS	890–660–460–300	0.59	300	1.7–1.2–0.8–0.5	1.63–1.11–0.82–0.48	Corona discharge	[111]
	5	a: Molded PDMS	570–496–403–314–184	–	550	1.2–1.0–0.8–0.6–0.3	1.17–0.94–0.71–0.54–0.23	Corona discharge	[107]

1: One-stage. 2: Two-stage. 3: Three-stage. 4: Four-stage. 5: Five-stage. a: Microfabrication process. b: Micromachining technologies. c: 3D printing. d: Simulation by FEM analysis. DRIE: Deep Reactive Ion Etching; ICPE: Inductively Coupled Plasma Etching; DFP: Dry film photoresistant; PDMS: Polydimethylsiloxane.

**Table A2:** Comparison between the key design and performance parameters of the MEMS and NEMS sensors for airborne PM detection found in the literature.

Type of Sensor	Particle Deposition	Sampling Method	Particle Size (μm)	Resonant Frequency (MHz)	Quality Factor (Q_f_)	Resolution (ng)	Sensitivity	LOD (μg m^−3^)	Integration Time (min)	Ref.
QCM	a: hydrogel film	μVI	PM2.5	11.98		830	–	8320	1	[102]
	a: heated grease film	CI	PM2.5	10		570	0.095 hz (ng cm^−2^)^−1^	15	1	[126]
	a: thin photoresist film	μVI	PM2.5	4.98	33,000	3.47 (calculated)	11 hz min^−1^	142	5	[106]
	a	μVI	PM1	4.98			5.1 hz min^−1^	52.33	10	[108]
SAW	a: glycerol film	μVI	PM1	147.24	190		7.46 hz min^−1^ per μg m^−3^			[109]
	e	–	PM1	894			7.5 khz per μg m^−3^			[129]
	e	–	PM2.5	262		0.21	262 Hz ng^−1^			[130]
	b	μVI	PM2.5	311	4500	0.17	93.96 hz min^−1^ per μg m^−3^	2	15	[96]
FBAR	b	–	PM2.5	1600		0.001	–	18	1	[128]
	b	μVI	PM2.5	600			–	2	10	[84,88,89]
	b	μVI	PM2.5	600			5	1		[97]
	b	μVI	PM2.5	600			7.05 hz min^−1^ per μg m^−3^	1	7	[98]
TPR	c	Vacuum chamber	PM0.1	1: 0.2–1.7	20,000–4400	0.115	50–300 hz ng^−1^		121 max	[136]
	c	Vacuum chamber	PM0.1	1: 60–20	11,000–4000	45 × 10^−4^ to 25 × 10^−4^	1.2–1.6 kHz pg^−1^		25	[134]
	c	CI	PM0.1	2: 5.3		–	42 hz pg^−1^		60	[133]
PCR	d	Air chamber	PM0.1	3: 16 × 10^−4^	155–300	12.1	8.31 × 10^−3^ Hz ng^−1^			[139]
	d	Air chamber	PM0.1	3: 2.6	480	8.9	11.15 × 10^−3^ Hz ng^−1^			[138]
	d	Air chamber	PM0.1	3: 44	1230	0.0048	8.33 Hz ng^−1^			[137,141]
	d	Air chamber	PM0.1	3: 44	1206	0.001	10 Hz ng^−1^			[140]
	d	Air chamber	PM0.1	4: 144.2	2100		32.75 Hz ng^−1^			[142]
	d	Micro fan	PM0.1	5: 221.5	1950 in air	5 × 10^−6^	36.51 Hz ng^−1^		15	[143]
	d	Micro fan	PM0.1	9.4 × 10^−3^				25	5	[144]
	d	Micro fan	PM0.1	6: 200	4700			5	6 seg	[145]
	d	Air chamber	PM0.1	7: 0.45	1200–1700	1.5 × 10^−6^	7220 kHz ng^−1^			[146]
Capacitive	e	–	PM1				65 zF			[151]
	e	Air pump	PM2.5–10				1.2 aF		10 ms	[150]
	b	μVI	PM2.5–10	4			–56.8 pF μg^−1^			[93]
	b	Air pump	PM2.5–10				0.48 zF			[152]
Corona discharge	f	μVI	PM0.1				8 × 10^–7^ pA (# cm^−3^)^−1^			[86]
	f	μVI	PM0.1				Comparable to *			[156]
	f	μVI	PM0.1				320 to 10^6^ # cm^−3^			[95]
	F	μCI	PM0.1				Comparable to **			[107]
Optical	f	NP condenser	PM0.1				0.21–10^5^ # cm^−3^		0.3 s	[158]
	f	Air pump	PM2.5					32.8	Real-time	[159]
	f	Air pump	PM2.5					10	Real-time	[160]
	f	μVI	PM2.5					2.55	Real-time	[94]

a: Adhesive film. b: Thermophoretic precipitator. c: Partial vacuum. d: Electrostatic sampling. e: Natural deposition. f: In-line detection. 1: Bulk resonant mode. 2: Extensional resonant mode. 3: Fundamental resonant mode. 4: Second resonant mode. 5: Damped nth resonant mode. 6: Fundamental lateral resonant mode. *: Commercial condensation particle counter. **: Aerodynamic Particle Sizer (self-sensing method).

**Table A3:** Comparison between the key design and performance parameters of the MEMS and NEMS sensors for airborne PM detection found in the literature.

Detection Principle		Characteristics	Sampling Method	Reaction Principle	Target Analyte	LOD	Integration Time (s)	Ref.
Continuous flow-based	Electrochemical	self-assembled monolayer (SAM)/multilevel air pillars	Hydrophilic-hydrophobic barrier/natural deposition	Nessler’s reaction	NH_3_	–	15	[180]
		modified Ca paste electrodes (CoPC-CPE)	PILS sampler	Oxidation of Dithiothreitol (DTT)	Urban oxidative activity	7 ng to 214 ng	180	[182]
		Capillary electrophoresis (CE)	–	Background electrolytes (BGE) dilution	So_x_/NO_3_/Cl/C_2_O_4_	160 nM/260 nM/190 nM/180 nM	25	[178]
		Glassy Ca electrodes	Modified 2,4-dinitrophenylhydrazine (DNPH)/Silica-gel cartridges	Aldehydes derivatization to form DNPH hydrazones	formaldehyde/acetaldehyde/2-propenal	9.5 µM/7.2 µM/9.2 µM	0.1	[179]
		Cu plate electrode	bioaerosol-in-hydrosol electrostatic sampler	Selective antibody-modified silicon nanowire transistors (SiNW-FET)	H3N2 airborne influenza virus	10^4^ viruses L^−1^	60–120	[181]
		Metallic coated microchannels	Microsampler	Commercial chemoresistive gas sensor	VOCs	–	150	[185,186]
	Optical	EW: λ = 640 nm Spectrometer and cellphone camera detection	Button air sampler	Latex immunoagglutination assay	H1N1/2009 virus	1 and 10 pg mL^−1^	300	[188]
		CCD fluorescence microscope detection	capture and enrichment micro-chamber	Fluorimetric immune adsorption reaction Ag85B antigens	M. tuberculosis	10^2^ cells mL^−1^	~4 h	[190]
		EW: λ = 365 nm	capture and enrichment micro-chamber	Loop-mediated isothermal amplification (LAMP)	*S. aureus*/*E. coli*/*P. aeruginosa*/*C. koseri*/*K*. pneumoniae	24 cells	~4 h	[191]
		EW: λ = 470 nm Photodiode detection	capture and enrichment micro-chamber	LAMP	*P. aeruginosa*	–	70 min	[193]
		Bioluminescence photodiode detection	Bioaerosol sampler	Adenosine triphosphate (ATP)/D-luciferin reaction	*B. subtilis*/*E. coli* JM110	concentration vs. intensity Linear growth	120	[198]
		EW: λ = 470 nm CCD fluorescence microscope detection	μCI- stained agar plate	Direct bioaerosol staining with SYBR green I dye	*S. epidermidis*	concentration vs. intensity Linear growth	10	[90]
		EW: λ = 510–550 nm CCD array camera	Biosampler	Direct bioaerosol staining with SYTO82 fluorescent dye medium	*E. coli*/*B. subtilis*/*S. epidermidis*	concentration vs. intensity Linear growth	25–250	[197]
Continuous flow-based	Spectroscopic	EW: λ = 514.5 nm	Sampling delivery gas system into an open microchannel	silver nanoparticles colloidal suspension to form SERS hot spots (AgNPs-SERS)	gaseous 4-aminobenzenethiol (4-ABT)	–	–	[199]
		EW: λ = 658 nm	Sampling delivery gas system into an open microchannel	AgNPs-SERS	2,4-dinitrotoluene (2,4-DNT)	1 ppb	120	[200]
		EW: λ = 648 nm	Sampling delivery gas system into a closed microchannel	AgNPs-SERS	4-ABT vapor	<2.5 pg	2.5	[201]
Droplet-based	Colorimetric	CMOS inverted microscope	Air-into-liquid sampler	Nessler’s reaction	NH_3_	–	–	[186]
		Fluorimetric microscope	Aerodynamic lens	fluorescent profile of *E. coli* produced with propidium iodide (PI)	*E. coli*	–	60	[194]
		High-speed camera	Filter-based and impinger sampling	Microdroplet freezing event from −5 °C to 35 °C	ice-nucleating particles (INPs)	–	60–90 min	[195,196]
		AW: λ = 540–365–608 nm Absorption spectrometer	μCI	MTB-barium complex	NO_3_/NH_4_/SO_4_	NA/0.256/11 ppm	60 min	[166]
Paper-based	Colorimetric	Scanned Images processed by image software	Filter-based personal sampler	Bathophenanthroline (Bphen) /Bathocuproine (BC)/ Dimethylglyoxime (DGM)	Fe/Cu/Ni	1 to 1.5 ug Linear range: 1 to 17 ug	–	[167]
		Scanned Images processed by image software	Filter-based sampling	1,5-diphenylcarbazide (1,5-DPC)	Cr	0.12 ug Linear range: 0.2–3.7 ug	–	[169]
		Scanned Images processed by image software	Filter-based sampling	Bphen/BC/DGM/1,5-DPC	Fe/Cu/Ni/Cu	Linear range: 1.1–10/0.15–6/1–10/1.5–8 ug	–	[172]
		Distance-based uPAD	Filter-based sampling	Bphen/dithiooxamide/DGM	Fe/Cu/Ni	<0.1 ug	–	[173]
		Cellphone image software application	unmanned aerial vehicle (UAV)	chrysoidine-G/dithiooxamide/ Bphen	Co/Cu/Fe	8.2/45.8/186.0 ng	–	[174]
		Cellphone image software application	UAV	Bphen/DGM/4-(2-pyridylazo) resorcinol (PAR)	Fe/Ni/Mn	Linear range: 170–1440/81–684/9.2–85 ng	–	[175]
Paper-based	Colorimetric	Cellphone image software application Field reaction kit	UAV	Chrysoidine-G/dithiooxamide/Bphen/PAR/ 1,5-DPC/DGM	Co/Cu/Fe/Mn/Cr/Ni	Linear range 8–81/45–458/186–1860/10–100/152–3810/80–800 ng	–	[176]
		Cellphone image software application	UAV	Graphene oxide nanosheetsBphen/dithiooxamide/DGM	Fe/Cu/Ni	16/5/10 ng	–	[177]
		Image software and distance-based uPAD	Filter-based sampling	DTT-oxidation	aerosol oxidative activity	Linear range: 0–75 ng and 5–25 ng	20 min	[168]
		Scanned Images processed by image software	Filter-based personal sampler	DTT-oxidation	aerosol oxidative activity	Linear range: 0–120 ng	30 min	[170]
	Electrochemical	Image software and modified Ca electrodes (Bi/ferricyanide)	Filter-based sampling	DGM/Bphen/BC/1,5-DPC	uPAD-Ni/Fe/Cu/Cr ePAD-Cd/Pb	uPAD-0.12 ug ePAD-0.25 ng	–	[183]
		modified Ca electrodes (Nafion/BiCSPE)	Ultrasonic personal sampler	1,10- phenanthroline/DMG	Cu/Fe/Ni/Cd/Pb	3.23/1.02/26.4/268.5/122.5 ng	–	[187]

EW: Excitation wavelength; AW: absorption wavelength.
